# Applicability of the Rapid Biophysical Profile in Antepartum Fetal Well-Being Assessment in High-Risk Pregnancies from a University Hospital in São Paulo, Brazil: Preliminary Results

**DOI:** 10.1155/2013/329542

**Published:** 2013-06-27

**Authors:** Jonathan Mamber Czeresnia, Edward Araujo Júnior, Eduardo Cordioli, Wellington P. Martins, Luciano Marcondes Machado Nardozza, Antonio Fernandes Moron

**Affiliations:** ^1^Fetal Growth Restriction Unit, Department of Obstetrics, Federal University of São Paulo (UNIFESP), Rua Carlos Weber, 956 Apartmente. 113 Visage, Vila Leopoldina, 05303-000 São Paulo, SP, Brazil; ^2^Department of Gynecology and Obstetrics, Faculty of Medicine of Ribeirão Preto, São Paulo University (FMRP-USP), Ribeirão Preto, SP, Brazil

## Abstract

*Objective*. To evaluate the clinical applicability of the rapid biophysical profile (rBPP), comparing results of the rBPP to umbilical cord pH values and Apgar scores. *Methods*. A cross-sectional study was conducted with 37 pregnant women from our high-risk service. All of them gave birth at our institution. rBPP was conducted up to 24 h prior to delivery and pH values were obtained from the umbilical vein immediately after birth. The mean and standard deviations for maternal age, gestational age at birth, pH values, and Apgar score in the 1st and 5th minutes after birth were calculated. An unpaired Student's *t*-test was applied to evaluate the correlation between these variables and rBPP scores of 2 and 4. *Results*. rBPP score of 2 was observed in 8 patients (21.6%) and score 4 was observed in 29 cases (78.4%). No patients received score zero. The difference between the Apgar scores of the rBPP score 2 and 4 was statistically significant (*P* < 0.01) while the same was not true with the umbilical cord pH (*P* = 0.08), even though the values tended to be lower in the rBPP 2 group. *Conclusion*. The rBPP is a fast and practical method of assessment of antepartum fetal well-being. Further studies, with a larger number of patients, are necessary to evaluate the applicability of the method.

## 1. Introduction

Various methods of evaluation of fetal vitality in high-risk pregnancies have been proposed in the past 30 years [1–3]. Among them is the fetal biophysical profile (FBP), proposed by Manning et al. [4] in 1980, which has stood out. It consists of the evaluation of 4 ultrasonographic criteria, 3 acute (fetal breathing movements, generalized fetal movements, and fetal tone), and one chronic (amniotic fluid index-AFI), and one cardiotocographic criterion (transient accelerations in response to fetal movements). Each criterion is given a score of 0 or 2 and the sum of all criteria yields the result. Scores of 8 and 10 are considered normal. In a study of 12,620 high-risk pregnancies, the perinatal mortality rate for normal values (≥8) was 0.652/1,000, while, for null scores, the mortality rate was 187/1,000.

Therefore, the BPP is an effective method for antepartum assessment of fetal well-being in high-risk pregnancies, but its applicability in the overcrowded and underfunded hospital is limited: a trained fetal sonographer, ultrasound apparatus, and a cardiotocographer are required to carry out the test, in addition to up to 30 minutes for the procedure to be accurately performed. The rapid biophysical profile (rBPP), proposed by Tongsong et al. [5], consists of the measurement of the amniotic fluid index (AFI) and observation sound-provoked fetal movement (SPFM). A subsequent study showed that the rBBP was effective in predicting adverse perinatal outcome with a 98.18% accuracy [6]. However promising the results may seem, there are no studies comparing the rBPP with umbilical cord pH values, considering the gold standard for testing fetal acidemia.

The goal of this preliminary study is to compare the rBPP scores with the Apgar score and umbilical cord pH values to verify the clinical applicability of the method in a highly demanding obstetric center such as the one in our institution.

## 2. Methods

A cross-sectional study with 37 pregnant women from our high-risk obstetrics ward was conducted from January through July 2012. The study was approved by the Ethics Committee in Research Committee of the Federal University of Sao Paulo (UNIFESP) and all patients agreed to be part of the study by signing an informed consent. The following criteria had to be met for inclusion in the study: single fetus, with no structural malformations detected after birth, and gestational age over 34 weeks.

The rBPP tests were performed by a single operator (JMC) up to 24 hours prior to delivery. The ultrasound machine used was a Voluson E8 (General Medical System, Healthcare, Zipf, Austria), equipped with a convex multifrequency transducer (RAB 4-8). All variables were obtained through real-time two-dimensional ultrasound. The AFI measurement was calculated by first dividing the uterus into four quadrants using the linea nigra for the right and left divisions and the umbilicus for the upper and lower quadrants. The maximum vertical amniotic fluid pocket diameter in each quadrant not containing cord or fetal extremities was measured in centimeters; the sum of these measurements was considered the AFI. Values over 5 cm were considered normal (score 2). The SPFM was obtained with a Toitu TR-30 Fetal Stimulator (Toitu Co., Ltd., Tokyo, Japan) by positioning the stimulator over the cephalic pole. The stimulus was applied for 3 seconds. The immediate detection of fetal movement by ultrasound was considered normal (score 2). A 2 mL of blood from the umbilical vein was obtained immediately after birth in 3 mL Becton Dickinson (BD Medical, Sao Paulo, SP, Brazil) plastic syringes. Samples were analyzed automatically 5 to 10 minutes after birth by the Radiometer ABL-5 (Diamond Diagnostics, Holliston, MA, USA) machine. The pH and pCO_2_ were calculated. The same person (JMC) obtained and analyzed the samples.

Data were transferred to an Excel 2007 spread sheet (Microsoft Corp., Redmond, WA, USA) and analyzed by the Statistical Package for Social Sciences (SPSS), version 15.0 for Windows (SPSS Inc., Chicago, IL, USA). The 37 patients were divided into two groups: normal (rBPP = 4) and abnormal (rBPP = 2). The mean and standard deviations for maternal age, gestational age at birth, pH values, and Apgar score in the 1st and 5th minutes after birth were calculated. An unpaired Student's *t*-test was applied to evaluate the correlation between these variables. The level of significance considered was *P* < 0.05.

## 3. Results

The rBPP was initially performed in 42 women, but 5 were excluded from the study because the umbilical cord blood was not drawn. Therefore, 37 patients were included in the study. Four of these had gestational diabetes mellitus; four had preeclampsia; two had chronic hypertension; two had pregestational diabetes mellitus; one had repeated urinary tract infection; one had HIV; one had chronic kidney disease. No pathological conditions were observed in the other 22 patients.

**Figure 1:**
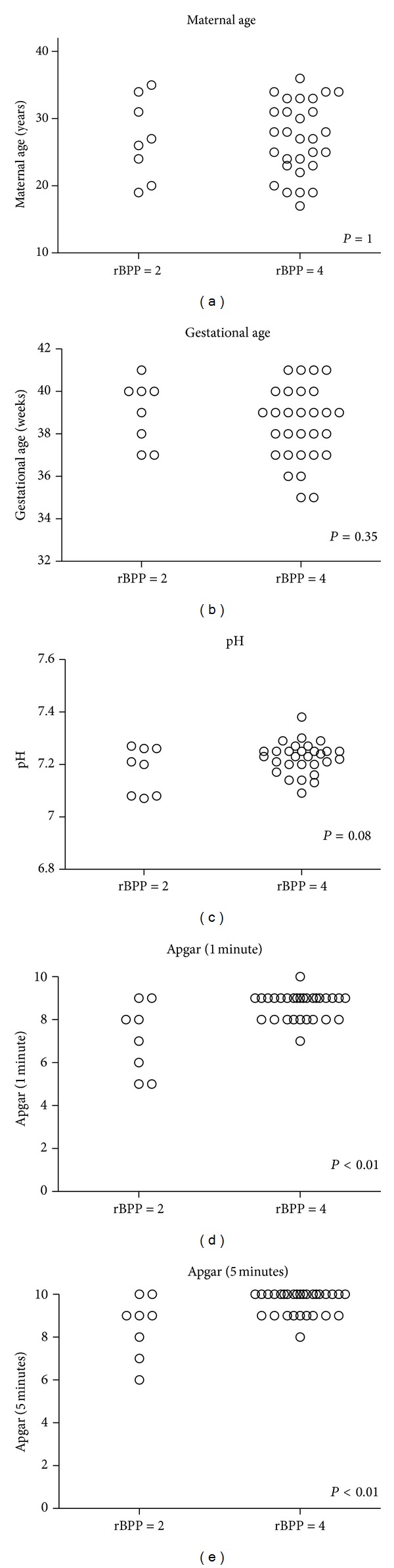
Correlation between normal (score 4) and abnormal (score 3) rBPP and maternal age (a); gestational age at birth (b); umbilical cord pH (c); Apgar score in the 1st minute (d); Apgar score in the 5th minute (e). rBPP: rapid biophysical profile.

Twenty nine of the 37 (78.4%) patients received rBPP score 4 (normal) and 8 (21.6%) received score 2 (abnormal). None received score zero. Mean maternal age was 27.0 ± 6.0 years, mean gestational age at the time of birth was 39 ± 1.5 weeks, mean pH value was 7.18 ± 0.09, and mean Apgar score at the 1st and 5th minutes after birth was 7.1 ± 1.6 and 8.5 ± 1.4, respectively. The difference between the Apgar scores of the rBPP groups was statistically significant (*P* < 0.01), while the same was not true with the umbilical cord pH (*P* = 0.08), even though the values tended to be lower in the rBPP 2 group (Table 1 and Figure 1).

## 4. Discussion

The BPP is a well-established method for antepartum fetal well-being evaluation. In a study of 86,955 fetuses published in 1999 by Dayal et al. [7], the fetal death rate after a normal BPP was 0.7–2/1,000. The main advantage of the method is its low false-positive rate, which means that it is extremely safe in case of a normal result. Despite being a good method, the applicability of the BPP in the overcrowded and underfunded hospital is limited: a trained and experienced fetal sonographer is required to carry out the test, in addition to up to 30 minutes for the procedure to be accurately performed. These limitations called for variations of the method, aiming to reduce the procedure time and the requirement of a trained examiner, without reducing its accuracy. The modified BPP associates AFI measurement and cardiotocography (CTG) and was demonstrated to be an effective method, with low false-positive rate and less time consumption than the classic BPP [8].

The rBPP, proposed by Tongsong et al. [5], associates SPFM with AFI measurement. The SPFM would reflect the neurologic state of the fetus at the time of the test (acute variable) and the AFI would reflect placental function (chronic variable). The time required to perform this test is approximately 2 minutes and it demands much less experience and skill from the examiner when compared to the BPP. In this pioneer study with 1,234 single-fetus high-risk pregnancies, the rBPP demonstrated higher sensitivity and specificity than intrapartum CTG for prediction of fetal suffering [5]. In a study with 330 laboring women by Tongprasert et al. [6], the rBPP was shown to have a 50% sensibility and 99% specificity in predicting abnormal neonatal outcome.

Phattanachindakun et al. [9] compared the classic BPP to the rBPP in predicting fetal well-being. Both tests were performed on 200 single fetus gestations between 30 and 42 weeks. The study demonstrated a strong correlation between the two tests. The BPP took 25.5 times longer to be carried out than the rBPP. Chousawai et al. [10] evaluated the applicability of the rBPP in predicting abnormal gestational results in fetuses with suspicion of growth restriction: in the 30 cases reported, the rBPP had 100% sensitivity and 89.7% specificity.

In our study, we compared cases of normal (4) and abnormal (0 or 2) rBPP scores to variables such as umbilical cord pH values and Apgar scores in the 1st and 5th minutes after birth. The pH is considered the gold standard for evaluation of fetal acidosis and closely correlates with classic FBP scores [11]. However, there are no studies comparing pH and rBPP values. When comparing Apgar scores between the normal (rBPP = 4) and abnormal (rBPP = 2) groups, statistical significance was observed, which proofs the rBPP as a good predictor of abnormal perinatal outcome in high-risk pregnancies. pH values were lower in the abnormal group, when compared to those of the normal group, but statistical significance was not observed, and we believe that because of small sample size. The SPFM was obtained with a Toitu Fetal Stimulator TR-30 (30–80 MHz), which is not expensive and easy to use.

We hereby present our preliminary experience with the rBPP in the assessment of antepartum fetal well-being in high-risk pregnancies. The rBPP, a fast and practical method, showed promising results that must be confirmed by studies with a larger number of patients.

## Figures and Tables

**Table 1:** Mean and standard deviation of maternal age, gestational age at birth, umbilical cord pH, and Apgar score in the 1st and 5th minutes in the normal (score 4) and abnormal (score 2) rBPP groups.

	rBPP 2 (*N* = 8)		rBPP 4 (*N* = 29)		*P *
	Mean	SD	Mean	SD	
Maternal age (years)	27.0	6.0	27.0	5.4	1.00
Gestational age (weeks)	39.0	1.5	38.4	1.7	0.39
pH	7.18	0.09	7.23	0.06	0.08
Apgar (1 minute)	7.1	1.6	8.7	0.6	<0.01
Apgar (5 minutes)	8.5	1.4	9.6	0.6	<0.01

rBPP: rapid biophysical profile; *P* values determined by unpaired *t*-tests.
